# Changes in sexual life experienced by women in Taiwan after receiving treatment for breast cancer

**DOI:** 10.1080/17482631.2019.1654343

**Published:** 2019-09-17

**Authors:** Yun-Chen Chang, Wen-Yu Hu, Yuh-Ming Chang, Shih-Che Chiu

**Affiliations:** a School of Nursing, College of Medicine, National Taiwan University, Taipei, Taiwan; b Department of Nursing, National Taiwan University Hospital, Taipei, Taiwan; c Cancer Center, Hsinchu Mackay Memorial Hospital, Hsinchu, Taiwan; d Department of Neurology, Hsinchu Mackay Memorial Hospital, Hsinchu, Taiwan; e Institute of Biochemistry, Microbiology, and Immunology, Chung Shan Medical University, Taichung, Taiwan

**Keywords:** Breast cancer, qualitative, sex life, sexual problem, in Taiwan

## Abstract

As the number of breast cancer survivors increases, these patients with sexual problems also increase. For breast cancer survivors, sexual problems are a common and painful experience. Although breast cancer survivors often encounter sexual problems, Taiwanese women are culturally conservative and patients rarely discuss sex problems with clinicians. In this study, we used qualitative methods to better understand the changes in sexual life and related care strategies for breast cancer survivors. Twenty interviews were conducted on clinical patients enrolled in hospitals that received breast cancer treatment. The data were analysed by performing a constant comparative analysis. Three themes emerged: the causes of changes in sexual life, internal response strategies and external response strategies. Ten subthemes were identified. Changes in sexual life in patients with breast cancer in this study included changes related to body image, influence of friends and family, age, genital problems, and illegal love of a partner. Breast cancer survivors can tolerate and regulate sexual life changes by adopting internal and external response strategies. Medical staff must be sensitive and must understand strategies for dealing with sexual life changes that may occur during cancer adjustment and how these strategies can help women’s well-being in the rest of their lives.

## Introduction

Breast cancer is the most common prevalent cancer for women in Taiwan (Ministry of Health and Welfare EY, Taipei, Taiwan, 2017). Because of advances in early detection and treatments, breast cancer mortality is decreasing. However, related studies have indicated that 41% of sexually active patients develop sexual function deterioration after breast cancer treatment (Alicikus et al., 2009). This is mainly due to loss of libido (80%), loss of interest in partners (54%) and sexual dissatisfaction (59%) (Alicikus et al., 2009). More importantly, sexual problems lead to serious emotional distress, including problems related to negative body image, anxiety, and depression (Boswell & Dizon, 2015).

Current mainstream treatments for breast cancer include surgery, chemotherapy, radiation therapy, and hormonal therapy. Among women younger than 50 years, total mastectomy is more likely to cause higher levels of depression and less satisfaction with body image than lumpectomy (Dorval, Maunsell, Descheˆ nes, & Brisson, 2000; Lasry et al., 1987). Chemotherapy and hormone therapy lead to changes in ovarian function (e.g., vaginal dryness, stomatitis, and postmenopausal function) (Avis et al., 2004; Milbury & Badr, 2013). Alopecia and loss of eyelashes are also common side effects of chemotherapy experienced by more than 90% of patients with breast cancer (Morris, Stinnett, & Woodward, 2011; Nozawa et al., 2013). Side effects of radiation therapy, including lymphedema, feeling very tired, pain, and fibrosis, may reduce libido. As mentions above, women with breast cancer have reported being less satisfied with their sex lives as well as feeling less attractive and less feminine in their appearance. In culturally conservative Taiwan, sexual problems among rare breast cancer survivors often do not actively seek advice from male doctors, and there are no clear guidelines for resolving problems during treatment and follow-up. In one study of patient experiences with sex issue, approximately 76% of the breast cancer participants confirmed that they have not ever asked medical staffs about problems with sex activities (Flynn et al., 2012). Therefore, medical staffs need to understand the extent of physical and psychological problems that may occur at each stage of cancer and during long-term follow-up so that appropriate support and assistance can be provided.

Sexual activity may also be affected by ageing; sexual needs or desire declines with age in almost all people (Kennedy, Martinez, & Garo, 2010). In some elderly people, especially women, sexual behaviour may be more concerned with non-verbal (e.g., physical intimacy) and verbal (e.g., emotional communication) methods than with sexual behaviour itself (Watters & Boyd, 2009).

After the treatment of breast cancer, the sexual life problem is urgently needed to be solved and understood. Recently, rarely qualitative research design of studies has addressed sexual dysfunction in women with breast cancer in Taiwan. Therefore, this study investigated the sexual problems of cooperative breast cancer survivors and hoped that medical personnel could actively explore their sexual life quality, address the root causes of their sexual life instability and their own coping strategies.

### Purpose

The purpose of this study was to apply one of the characteristics of qualitative research, namely the trust between researchers and participants that is necessary for revealing the true perceptions of patients, integrated with the concepts of grounded theory in order to explain coping strategies and changes in the sexual behaviour of breast cancer survivors after treatment. In accordance with the core goal of grounded theory, the current research was conducted on the basis of the following question: “What are the important phenomena for the research population?”

### Methods

We employed a qualitative research method by conducting semistructured interviews with breast cancer survivors from May 2017 to February 2018. This research used a grounded theory approach which was part of a larger qualitative study on the sex lives of women who undergo breast cancer treatment.

#### Participants

The participants were 20 women with breast cancer selected through theoretical sampling who ranged in age from 36 to 64 years at the time of interview (mean age, 48.5 years) and who were all in relationships with men. The time from diagnosis to interview ranged from 2 months to 5.7 years (mean, 1.3 years). The breast cancer stages in this subset ranged from stage I to stage IV. The sample size was determined based on data saturation. To protect the privacy of all subjects were transcribed using pseudonyms (Table I).

**Table I.** Baseline characteristics of participants.

Patient	Age	Cancer Stage	Religious beliefs	Surgery	Current Treatment^a^	Relationship status
Lucy	50	IIIA	No	Mastectomy	CT	Married
Fiona	62	IA	Buddhism	Mastectomy	F/U	Divorced; Partnered
Hellen	47	IIB	Taoist	Mastectomy	TT	Married
Cherry	41	IIB	Buddhism	Lumpectomy	RT	Married
Jane	52	IIA	Buddhism	Mastectomy	CT	Married
Lena	52	IB	No	Mastectomy	HT	Died; Partnered
Carol	54	IIIC	Buddhism	Mastectomy	TT	Divorced
Christine	41	IA	Catholicism	Lumpectomy	HT	Married
Abby	47	IA	No	Mastectomy	HT	Married
Kelly	64	IA	Taoist	Lumpectomy	RT	Married
Rebecca	36	IA	Buddhism	Mastectomy	RT	Remarried
Sandy	52	IIA	Taoist	Lumpectomy	HT	Married
Sharon	43	IA	Taoist	Lumpectomy	F/U	Married
Teresa	51	IV	No	Mastectomy	TT	Married
Una	47	IIIA^b^	No	Lumpectomy	RT&HT	Partnered
Wendy	44	IIA	Buddhism	Lumpectomy	HT	Married
Zera	48	IIB	Buddhism	Mastectomy	RT & TT	Married
Vicky	53	IA	Buddhism	Lumpectomy	CT	Married
Tracy	43	IIIA	Taoist	Lumpectomy	CT	Married
Susan	43	IA	Taoist	Lumpectomy	CT	Married

^a^CT, Chemotherapy; HT, Hormone therapy; RT, Radiotherapy; TT, Target therapy; F/U, Follow-up

^b^Pre-operation, Clinical stage

#### Data collection

Corbin and Strauss (Corbin & Strauss, 2014) proposed criteria that should be met by researchers when applying grounded theory, and these include retrieving data from the technical literature, stimulating the induction process during research, remaining sensitive to what patients say and do, and identifying the main categories of the patient experience.

According to Glaser (1992) (Glaser, 1992), researchers applying grounded theory must select an area of interest and problem to address and that there are no absolute right or wrong decisions in this respect. Accordingly, interviews conducted in the present study focused on obtaining responses from patients by presenting them with the following questions and prompts pertaining to their sexual behaviour:

1. What is the main reason for changes in your sexual life?
2. Please express your feelings regarding the changes that you have experienced in your sexual life after treatment.
3. How are you dealing with changes in your sexual life after treatment?

Each interview lasted between 30 minutes and 1 hour and was conducted at the hospital’s Cancer Resource Centre. Data collection ceased when investigators determined that interviews did not produce new themes and continued until saturation was reached.

## Ethical approval

The Mackay Memorial Hospital Institutional Review Board approved (17MMHIS062e) this meaningful study. Eligible participants were informed of the research purpose before providing written informed consent. They were also assured of confidentiality and anonymity.

## Data analysis

Glaser (1998) (Glaser, 1998) suggested that memorandums be freeform and handwritten, advising against the use of computer programs or narratives because such approaches hinder the development of grounded theory. Therefore, the interviewer (YC) transcribed interviews verbatim by using Microsoft Excel® within 1 day after the interview. The application of grounded theory is generally regarded as useful when studying psychosocial situations (Baker, Wuest, & Stern, 1992). Two researchers (YC and CC) analysed data in four stages and applied the constant comparative method (Hallberg, 2006) at all stages. In the first stage, the transcripts of patient responses were deconstructed into paragraphs and typed line-by-line in the Excel file and keywords were identified. Keywords and sentences were then assigned labels in a process called “open coding” and then compared with each other. In the second stage, the Excel filter function was used to manually code each transcript, and their properties were used for classification into conceptual categories. In the third stage, to group categories into subthemes, two researchers (YC and CC) discussed cross-case texts on the basis of constant comparison. The aim of this coding process is to clarify how subthemes that appear are related to the initial theme and then compare how initial themes are integrated. This integrated main theme is considered to be the centre of the phenomenon; through theoretical comparison of subthemes with the central axis, “axis coding” was used to discern similarities and differences among attributes. Finally, the main core theme is the focus when interpreting the narrative of women with breast cancer and the significance of their behavioural changes. To achieve our aim, patients were subsequently invited to review data and determine whether the content was correct or needed to be supplemented.

## Findings

Change et al. (2018) (Chang, Chang, & Chiu, 2018) conducted a systematic literature review that revealed that factors affecting the sexual life of patients with breast cancer include age, religious beliefs, treatments, and communication with sexual partners. However, the aforementioned research process produced results that revealed relatively broad themes. The results of interviews with 20 participants were derived by applying the constant comparative analysis method. In this process of continuous comparison, the relationship between concepts emerges and constitutes a theoretical model. The final grounded theory, presented in Table II, details changes that patients perceived in their sexual life (core theme), including the development of a negative body image, decreased sexual desire or genital problems, influences from family members, their age, and infidelity of their partners. Internal and external response strategies have emerged as useful for many patients and are therefore included in grounded theory. Grounded theory outlines the 10 subthemes that might influence sexual behaviour.

**Table II.** Major themes and subthemes.

Themes	Subthemes
Reasons for Sex Life Changes	Change in body image due to surgery
	Change in body image due to chemotherapy
	Sexual desire decreased or genital problems due to Medication
	Change in sex life influenced by other relatives
	Change in sex life influenced by age
	Partner’s illegal love affair
Internal Response Strategies	Religious beliefs
	Partnered Support
External Response Strategies	Change of external image
	Communication with husband or partner

### Reasons for sex life changes

#### Change in body image due to surgery

The most general themes were of dissatisfaction with the surgery in term of persisting pain or scarring. One of the participants said:

I had engaged in sex approximately twice per month before surgery but the frequency changed to once a month for the reason of wound pain. (Lucy)

The other said:

I exhibited an overall reduction in sexual desire after surgery. Sometimes my husband wanted to have sex, but I refused due to wound pain. (Cherry)

Breast mastectomy entirely and scars after surgery leave patients with less self-confidence and thus affect sexual desire. One of the patient expressed as follows:

My breast was removed entirely (wry smile), I had suddenly lost my sex desire. (Jane)

The technique of sewing by the doctor is very important for the patient. One patient indicated the following:

… (Clothes pulled open) the scar is black and sunken … the wound is like being bitten by a dog, making me fear looking in the mirror. (Carol)

Nipple represents female symbol. Whether the mastectomy retains the nipple is crucial for patients with breast cancer.

… the doctor had performed to remove the nipple; this was a heavy blow to me. (Kelly)

The other patients said:

I had asked the plastic surgeon whether my nipple would be spared … surgery completed; my nipple looked like “dead raisins”. (Rebecca)

I thought that my nipple had shrunk a little and that the color was darker and different to the others nipple after surgery. (Sandy)

#### Change in body image due to chemotherapy

Chemotherapy-caused alopecia was among the most annoying adverse effects; these women with breast cancer commonly felt particularly sad due to esthetic damage and often consequently lose self-confidence (Ghizzani, Bruni, & Luisi, 2018).

The bald head affects my libido. When my hair grows after treatment, I may have sex with my husband as before. (Tracy)

My husband seemed unable to accept that I do not have hair; he felt that I do not have all the qualities of woman and no longer attractive. (Cherry)

My hair was falling out, leaving me with a relatively male appearance. In addition, many large patches of acne over my face, rotting nails, and pain around my feet were also noted. (Jane)

#### Sexual desire decreased or genital problems due to medication

Chemotherapy-associated menopause was extraordinarily related to sexual dysfunction and inactivity after treatments (Lee, Kim, & Jeon, 2015). One patient said as follows:

I seemed to be having menopausal symptoms and had no libido. (Hellen)

More importantly, patients are considered to have low immunity during chemotherapy and are vulnerable to sexual life.

Docetaxel (Taxotere®) drugs cause menopausal symptoms in women. I was afraid of vaginal mucosa damage, and thus my libido was always low at that time. (Sharon)

Another patient has a different perspective:

… chemotherapy made me tired and reduced my libido. In addition, my body was so poisonous, so I did not want to have any contact with my husband. I was afraid that it would affect him. (Christine)

… because of “this poison”, my vaginal itched, and thus I dared to have sex. When I had sex (laughing) one time, I am itched afterward. (Jane)

Most of the East Asian women’s personality was more conservative and traditional. Most women regard husband as a “God”. Although chemotherapy decreases libido in patients with breast cancer, many patients remain willing to cooperate with their spouses.

During chemotherapy, I felt depressed, vaginal dryness, and late-onset menopause which reduced my libido. But I am very embarrassed to consult a male doctor. Because of chemotherapy induced white blood cell count decreased, I worried that having sexual intercourse would lead to vaginal mucosa damage and infection. Even wishing to escape, I still cooperated with my husband when he asked to have sex. (Sandy)

The side effects of hormone therapy also affect patients with breast cancer. Patients said as follows：

… taking Tamoxifen (Nolvadex) had caused vaginal secretion and discharge. However, I did not talk to my attending physician for the same reason as perineum discomfort noted after treatments. (Sandy)

I was taking Tamoxifen which made me had hot flashes and very drying in vaginal, thus sexual intercourse was painful. (Abby)

#### Change in sex life influenced by other relatives

Reactions from family members and friends affect changes in the sex lives of patients with breast cancer. Family members and friends provided information that is not necessarily correct to the patient. However, patients have changed their sexual life patterns because they believed in them. One of them said：

My mother-in-law often told me not to have sex or I would die soon. (Carol) Another patient said that my friend had warned me that I would become overly excited and induce my cancer cells to become activated, if I had sex … laughs … (Fiona)

Sexual activity during chemotherapy is also considered a life-threatening event; it can even cause death.

My friend told me that having sex is too dangerous and that abstaining from sex after chemotherapy is recommended … (Abby)

There is a more serious statement. My sister had told me not to have sex because it would cause me to die early. (Zera)

Especially, after the chemotherapy of breast cancer survivors, the odour changes of the excrement are produced, which also affects the patient’s sexual life.

I did not have sex after chemotherapy because my urine had a drug-like aroma, so I did not want to have sex. However, artificial blood vessels (port-A) can also affect my feelings. (Lena)

Some patients are worried that engaging in sex life will affect the position of artificial blood vessels, because engaging in sex was considered a fierce sport.

I do not think I can have “excessively vigorous exercise” (means sex activities) during chemotherapy because I worried about transposition of artificial blood vessels. I went to say that I have to resume sexual activity after treatment. (Susan)

#### Changes in sex life influenced by age

Age is also one of the factors that affect sex life. The more mature a woman is, the lower the demand for sex. On the other hand, after giving birth, sexual desire will also decrease.

I seldom had sex, not because of the treatment but because of my age. (Teresa)

My sexual desire has gradually declined due to the birth of a second child and aging (Vicky, 42 years old).

#### Partner’s illegal love affair

A partner’s illegal love affair is a great blow for a woman, causing great psychological pressure, depression, and changes in sex life. Even women thought that men’s genitals are very dirty, so women do not want to have sex with their partners. There is a patient with this experience.

My husband had had an affair so I had depression. I felt that having sex with husband was unpleasant. So, I said “No” when my husband asks for sex. (Carol)

### Internal response strategies

#### Religious beliefs

If a patient has a religious belief, it can bring peace of mind and improve the quality of life. Religious beliefs influence the reactions of individuals to sex.

I attended the nightly sermon in the hospital every evening; during the sermon, she would calm down and follow Jesus. Other things will not think about it. (Lucy)

I had started to read the Buddhist scriptures that made her feel calmer after cancer diagnosed. (Vicky)

#### Partnered support

When partnered support is more powerful, patients face the disease with decreased psychological pressure and have better prognoses (Goodwin, Hunt, Key, & Samet, 1987).

… husband treated me well after I became diagnosed. He was very supportive, accompanied me to the hospital when I went for treatment, and often gave me verbal encouragement. He was my “potent pill”. (Sandy)

### External response strategies

#### Change of external image

Treatment might change a patient’s body image; there are many methods to counter these adverse effects.

My friends had told her must use scar gel because if I did, when my husband saw the wound after breast surgery, he would have sexual desire for me. (Wendy)

One of the representative of patients who have undergone a total resection, so her contingency measures are as follows:

I would never expose myself to my husband in the nude and I always wear gauze to cover one side of my body in the front of my partner for maintaining my sense of mystery. (Fiona)

I felt nauseous when I looked at the black surgical scar, so I wore a dress when engaging in sex. (Vicky)

#### Communication with husband or partner

Couples’ positive communication is considered as a factor affecting emotional intimacy and sexual relationship satisfaction. Some patients say their experience in communication.

I asked my boyfriend regarding sexual position of engaging in sex. He would kiss the mastectomy scar and told me that he loved me but not my breast. (Lena)

My husband told me that life is more important than beauty and ugliness. (Zera)

## Discussion

Changes of the participants’ body image explained the negative psychological and physiological impacts of surgery and adjuvant chemotherapy, especially the first year after surgery (Ussher, Perz, & Gilbert, 2012). Evidence suggests that regardless of the kind of treatments received, body image will be disturbed and often associated with other problems such as those related to sexual dysfunction in the population of women with breast cancer (Paterson, Lengacher, Donovan, Kip, & Tofthagen, 2016).

East Asian female culture is more conservative than Western countries, and the patient’s attending physicians are usually men. Therefore, patients are less talkative with doctors about sexual health problems. Although previous qualitative studies have investigated the experiences of breast cancer survivors in terms of sexual function and fertility (Barthakur, Sharma, Chaturvedi, & Manjunath, 2017; Perz, Ussher, & Gilbert, 2014), this study was to understand the reasons for sex life changes and adopt coping strategies. Internal responses strategies can be based on religious beliefs that increase perceived health self-efficacy, functional well-being. Shaw et al. (2007) and the ability to deal with situations (McCrae, 1984). Pious religious activities are reported to be one of the most common coping responses to patients with cancer (Sherman & Simonton, 2001). Some patients in this study felt greatly supported by their religious beliefs, and women in more religious countries may rely even more on this form of coping (Jassim & Whitford, 2014; Ursaru, Crumpei, & Crumpei, 2014).

Supplying social support systems of others can influence the quality of patients’ lives and may even reduce mortality risk (Brown, Nesse, Vinokur, & Smith, 2003). Social support systems provide a patient with verbal encouragement and physical support.

Many researchers have stated that external appearance dissatisfaction has a negative emotion on sexual pleasure. Therefore, external response strategies can be adopted, including a change in one’s external image. Many patients indicate that the nipple is a female symbol. The results of past psychology studies showed that approximately 97.6% have high satisfaction with nipple preservation after surgery, especially for young patients (Petit et al., 2006). The more positive body images the more sexual self-confidence (Yamamiya, Cash, & Thompson, 2006), intercourse frequency (Trapnell, Meston, & Gorzalka, 1997), and sexual arousal in women (Purdon & Holdaway, 2006). Despite one participant of this study talk about that to have sex will be too excited to stimulate cancer cell growth and one patient indicated suggest wearing a condom during intercourse due to the toxicity of semen in a pilot study. However, previous studies have shown that sexual intercourse improves physical and mental health. For example, sex can reduce pain (Hambach, Evers, Summ, Husstedt, & Frese, 2013) and the risk of cardiovascular disease (Hall, Shackelton, Rosen, & Araujo, 2010). One or two times per week can increase an individual’s antibody concentration by 30% and strengthen the immune system (immunoglobulin A, IgA). Charnetski & Brennan (2004) and sexual activity stimulates the brain to release sexual reward-related neurochemical substances called opioid and oxytocin (Pfaus et al., 2012). However, patients with breast cancer may still have sex during treatment, but should pay attention to avoiding the problem of vaginal infection.

Another external response strategy is to communicate with the husband or partner. Manne indicated that effective communication is vital for couples (Manne & Badr, 2008). A couple’s level of communication was considered a factor influencing emotional intimacy, sexual satisfaction, and relationship satisfaction (Yoo & Bartle-Haring, 2014). Sexual activities changes have less of an impact on older patients; previously, the literature suggests that sexual desire declines with age (Granville & Pregler, 2018). The findings of this study found that female sexual desire began to decline between the ages of 42 and 50. However, in this study, there are also 63-year-old women who are engaged in sex life. Therefore, age is not the main factor affecting sexual life but health (Gewirtz-Meydan et al., 2019). Most notably, truly meaningful relationships do not force genital intercourse. Intimacy can be maintained by holding hands, hugging, caressing, and kissing. In fact, mutual care, communication, and companionship are the primary means of maintaining long-lasting relationships.

## Limitations

Several limitations of this research warrant consideration. First, this study was cross-sectional in design. Further studies are recommended to be longitudinal in design to notify the researchers about the lifetime effects of positive and negative coping strategies after breast cancer treatment. Second, sample size only 20 subjects which limit the generalizability of the results. Third, all the participants were entirely heterosexual, different age, and composed of Asian women. Studies should distribute other sexual orientations, younger women (<40 years old), other ethnic groups and perceptions of breast cancer patients’ husbands in the future.

## Implications for practice

The implications of this study are threefold. First, regarding the patients, Taiwanese women influenced by their conservative culture can bravely express the problems that they experience concerning changes in their sexual behaviour and actively seek help. Second, the family and friends of patients can provide patients with appropriate counselling and support. Third, Taiwanese doctors, especially male doctors, can be informed of the importance of paying attention to changes in the sexual life of female breast cancer survivors after treatment. Meanwhile, considering patient experiences and implementing suitable response strategies can improve the understanding that medical staff have of the experiences of breast cancer survivors, which will help them to actively solve problems encountered by these patients. Topics concerning patient sexual life should be integrated as much as possible into the daily standard operating procedures of health education in order to improve the quality of life of breast cancer survivors regarding their experiences of intimacy.
